# Biofilm producing plant growth promoting bacteria in combination with glycine betaine uplift drought stress tolerance of maize plant

**DOI:** 10.3389/fpls.2024.1327552

**Published:** 2024-02-09

**Authors:** Tahira Yasmeen, Muhammad Saleem Arif, Mohsin Tariq, Sadia Akhtar, Afira Syrish, Waqas Haidar, Muhammad Rizwan, Muhammad Iftikhar Hussain, Ajaz Ahmad, Shafaqat Ali

**Affiliations:** ^1^ Department of Environmental Sciences, Government College University Faisalabad, Faisalabad, Pakistan; ^2^ Department of Bioinformatics and Biotechnology, Government College University Faisalabad, Faisalabad, Pakistan; ^3^ Department of Plant Biology & Soil Science, Universidade de Vigo, Vigo, Spain; ^4^ Department of Clinical Pharmacy, College of Pharmacy, King Saud University, Riyadh, Saudi Arabia; ^5^ Department of Biological Sciences and Technology, China Medical University, Taichung, Taiwan

**Keywords:** oxidative stress, antioxidants, drought tolerant rhizobacteria, photosynthetic pigments, moisture stress levels

## Abstract

**Introduction:**

The escalating threat of drought poses a significant challenge to sustainable food production and human health, as water scarcity adversely impacts various aspects of plant physiology. Maize, a cornerstone in staple cereal crops, faces the formidable challenge of drought stress that triggers a series of transformative responses in the plant.

**Methods:**

The present study was carried out in two sets of experiments. In first experiment, drought stress was applied after maintaining growth for 45 days and then irrigation was skipped, and plant samples were collected at 1^st^, 3^rd^ and 6^th^ day of drought interval for evaluation of changes in plant growth, water relation (relative water content) and antioxidants activity by inoculating indigenously isolated drought tolerant biofilm producing rhizobacterial isolates (*Bacillus subtilis* SRJ4, *Curtobacterium citreum* MJ1). In the second experiment, glycine betaine was applied as osmoregulator in addition to drought tolerant PGPR to perceive modulation in photosynthetic pigments (Chlorophyll a and b) and plant growth under varying moisture stress levels (100, 75 and 50% FC).

**Results and discussion:**

Results of the study revealed upsurge in root and shoot length, fresh and dry biomass of root and shoot besides increasing chlorophyll contents in water stressed inoculated plants compared to uninoculated plants. Glycine betaine application resulted in an additional boost to plant growth and photosynthetic pigments, when applied in combination with bacterial inoculants. However, both bacterial inoculants behaved differently under drought stress as evident from their biochemical and physiological attributes. Isolate SRJ4 proved to be superior for its potential to express antioxidant activity, leaf water potential and relative water contents and drought responsive gene expression while isolate MJ1 showed exclusive increase in root dry biomass and plant P contents. Though it is quite difficult to isolate the bacterial isolates having both plant growth promoting traits and drought tolerance together yet, such biological resources could be an exceptional option to be applied for improving crop productivity and sustainable agriculture under abiotic stresses. By exploring the combined application of PGPR and glycine betaine, the study seeks to provide insights into potential strategies for developing sustainable agricultural practices aimed at improving crop resilience under challenging environmental conditions.

## 1 Introduction

Drought is a phenomenon caused by imbalance of water in soil resulting in food security concerns directly while health issues and population displacement indirectly. The global demand for food crops is anticipated to increase by 50-60% between 2019-2050 due to ever increasing population (Falcon et al., 2022). Due to the increase in average global temperature, light intensity and reduced rainfall the environment is becoming more unpredictable with frequent drought occurrence (Seleiman et al., 2021). This rapid climate change has severely affected the agriculture sector. Drought stress severely hampers plant growth and productivity by modulating numerous morphological, bio-chemical, and physiological responses (Hussain et al., 2020; Gui et al., 2021). Drought stress induces oxidative stress by up-regulating antioxidant enzymes activities such as that of ascorbate peroxidase (APX), glutathione peroxidase (GPX), superoxide dismutase (SOD) and catalase (CAT) etc. (Hussain et al., 2018; Sohag et al., 2020).

Although the crops are being developed involving genetic engineering trials, adjustment of crop calendar and resource management practices to mitigate moisture stress, however, these approaches are laborious, time consuming and expensive delivering output with less precision (Fadiji et al., 2022). Application of microbes on the other hand, is potentially competitive, suitable and environment friendly approach to improve plant tolerance against drought stress (Kang et al., 2014). A number of rhizospheric microbes have ability to produce plant growth regulating substances (Gujral et al., 2013; Grover et al., 2014; Dutta et al., 2015), secret exopolysaccharides (Igiehon et al., 2019), solubilize and mobilize nutrients, synthesize 1-aminocyclopropane-1-carboxylate (ACC) deaminase (Ojuederie et al., 2019), sequester iron (Etesami and Maheshwari, 2018) and induce systemic resistance (Olanrewaju et al., 2017). These microbes are capable of developing symbiotic relationships with the host plant. Plants provide photosynthetically fixed carbon to these microbes and in rejoinder microbes provide tolerance against biotic and abiotic stresses, improve acquisition of nutrient, enhance photosynthetic and water use efficiency and eventually facilitate better plant growth (Nihorimbere et al., 2011; Huang et al., 2014). Symbiotic interaction of efficient drought tolerant PGPR as bioinoculant prior to the drought onset could help plants for better response to subsequent drought and succeed to beat low productivity issues in drylands (Kaushal and Wani, 2016; Shirinbayan et al., 2019).

Besides PGPR, some compatible solutes like glycine betaine are linked with abiotic stress tolerance in transgenic plants (Chen and Murata, 2011). The two main and precise roles of glycine betaine are osmotic adjustment and cellular compatibility besides their involvement in ROS (reactive oxygen species) scavenging, macromolecules protection, and as carbon and nitrogen reservoir (Umezawa et al., 2006). However, accumulation of compatible solutes in lower extent is one of the considerable limitations in transgenic plants under abiotic stress. At such low concentration, compatible solutes are unable to aptly contribute to osmotic adjustment. Hence, application of exogenous glycine betaine as osmoregulators could help plants to withstand drought stress. Alleviation of drought stress in maize plants by applying exogenous glycine betaine has previously been reported by different research groups (Shafiq et al., 2021; Shemi et al., 2021). However, there is limited information about the response of drought stressed plants to combine application of glycine betaine and bacterial inoculants. Therefore, application of synthetic glycine betaine to assist plants under abiotic stress condition in the presence or/and absence of PGPR may be exploited to better understand their different aspects of functionality.

The aim of the present investigation was to isolate native drought tolerant PGPR from rhizosphere of water stressed plants, to evaluate their plant growth promoting (PGP) activities under drought stress and to evaluate how these selected microbes influence maize plant growth and physiology in response to drought stress in the presence of glycine betaine. Maize is one of the essential and widely grown cereals and among the biotic and abiotic stresses, drought stress is considered the main limitation for maize production, globally (Oumarou et al., 2019; Hunter et al., 2021). With a progressive increase in the human population, food demand is consistently increasing, and maize can be utilized as a food supplement to overcome the food scarcity challenges. Herein, we report that the selected isolates have great potential to be used as biotechnological tool for dryland agricultural systems and the option of stress alleviators like glycine betaine co-application with bacterial inoculants can produce promising results in term of plant growth and productivity.

## 2 Materials and methods

### 2.1 Bacterial isolation & screening for drought tolerance

Maize plant rhizospheric soil samples were collected from 32.9425°N, 73.7257°E (Jhelum), 30.8138°N, 73.4534°E (Okara) 32.9328°N, 72.8630°E (Chakwal) and 32.2955°N, 72.3489°E (Khushab) for isolation of drought tolerant plant growth promoting rhizobacteria (PGPR). To isolate viable bacterial colonies, serials dilution technique was employed using sterile normal saline solution (0.89% NaCl) followed by spread plate method (Islam et al., 2014a). Nutrient agar media was used besides Kings B media to get diversity of bacterial isolates. Out of 40 purified colonies (based on colony morphology i.e., shape, size and color), twelve bacterial colonies with different morphological characters were screened for their drought tolerance and PGP potential.

Morphologically diverse purified bacterial colonies were inoculated to autoclaved Kings-B broth having different concentrations of polyethylene glycol (PEG 6000) i.e., 0%, 1.67%, 4.16%, 7.30%, 10.85%, 15%, and 20% (Michel and Kaufmann, 1973). Inoculated media was incubated for 24 hours under constant shaking (120rpm) at 28°C to raise cultures of selected bacterial isolates. Bacterial culture was harvested and absorbance at 600 nm was recorded using a double beam UV-visible spectrophotometer. The polyethylene glycol was used to assess drought potential of isolated bacteria and the process was repeated thrice to acquire distinct drought resistant bacteria.

### 2.2 Physiological and biochemical characterization of drought tolerant rhizobacteria

Bacterial isolates those were found most resistant to drought stress were further evaluated for their biochemical characteristics and PGP characters including phosphates solubilization, auxin production, ACC-deaminase activity, siderophore production and root colonizing ability. Isolation and screening of drought tolerant phosphate solubilizing bacteria was carried out according to pore plate and streak plate method (Islam et al., 2014a; Islam et al., 2014b; McLellan et al., 2009) using Kings-B media having tricalcium phosphate (TCP) as main source of phosphorus (P) as described by Grover et al. (2013). A single fresh rhizobacterial culture colony was inoculated and incubated at 30°C for a week and observed for clear halo-zone formation around colonies. Phosphate solubilization ability of selected strains was evaluated at different PEG levels (0%, 1.67%, 4.16%, 7.30%, 10.85%, 15%, and 20%). The colonies with larger clear zones were further purified. The tendency of isolates to solubilize TCP was also verified by growing selected drought tolerant rhizobacteria in Pikovskaya broth (pH 7.0) (Pikovskaya, 1948) for 7 days and observing absorbance at 882 nm. The actual value was computed from the equation derived from the standard curve. Decrease in pH of solutions after 3 and 7days was also measured. All the physiological and biochemical analysis were performed in triplicates.

Aminocyclopropane-1-carboxylic acid (ACC) deaminase activity of selected strains was assessed at different PEG levels. Refreshed strains were grown using trypticase soya broth at 30°C (150 rpm; 24 hr). Cell pellets were obtained by centrifugation and washed with Tris-HCl (0.1M; pH 7.6). Pellets were suspended in DF minimal salt medium having ACC (3 mM) as sole nitrogen source and different concentrations of PEG 6000. Incubated at 30°C (150 rpm; 36 hr). Cell free extract was utilized to determine enzyme activity as per illustrated by Saleh and Glick (2001). The sum of α-ketobutyrate released by hydrolysis of ACC was recorded at 540 nm using standards of α–ketobutyrate (10 -500 μMol) (Penrose and Glick, 2003). Lowry method used to measure protein concentration of suspension (Lowry et al., 1951).

The selected drought resistant rhizobacterial strains were also screened for production of Indole acetic acid (IAA) at different concentrations of PEG following Gordon and Weber (1951) methods. Briefly, fully grown cultures (with and without L-tryptophan) were centrifuged (6000 rpm/10 min) and 1mL of supernatant was dissolved with 2mL of Salkowski’s reagent (1mL 0.5 M FeCl_3_; 30 mL conc. H_2_SO_4_; 50 mL distill water). The absorbance of pink color was measured at 530nm (Sheng et al., 2008). The standard curve for standards of IAA was prepared and IAA content of the samples were calculated.

The Siderophore production assay of the isolated strains was carried out by spot inoculating test on chrome azurole S (CAS) agar plates incubated in dark at 28°C for 96-120 hrs. Orange holo-zone formation around the colony was considered as positive for siderophore production (Schwyn and Neilands, 1987).

Bacterial exopolysaccharide production was estimated by growing bacterial strains on RCV mineral medium enriched with mannitol, glucose or sucrose (Ashraf et al., 2004). Biofilm secretion was examined on polyvinyl chloride (PVC) surface by a microtiter plate assay with modified protocol of Fujishige et al. (2006). The bacterial cultures were grown in YEM broth upto an optical density 2.0 at 600 nm, centrifuged at 10,000 rpm for 1 min to form pellets, and further diluted to 0.2 at 600 nm. Bacterial cell suspension (150 µL) was loaded in a PVC plate of 96-wells. Sterilized YEM broth was loaded as the control. The plates were wrapped with plastic covers and placed for incubation at 28 ± 2°C for 48 hr. Later on, the medium was wiped out and washed with autoclaved distilled water. The plates were left to dry, and 0.001% crystal violet (150 µL) was put in the wells for 20 min. Later on, the additional dye was drained, and the wells were cleaned with autoclaved distilled water. The absorbed stain was solubilized with of 95% ethanol (150 µL) and absorbance at 570 nm was noted to measure the solubilized dye.

Root colonization potential of the selected drought tolerant rhizobacteria was investigated under different moisture conditions (0-20% PEG levels). The sterilized cotton was moistened and sterilized maize seeds were sown on these plates. The seeds were also inoculated with the selected rhizobacterial isolates and plates were stored at 25°C. After 5 days, 0.4 g root tips were excised and put in 10mL sterilized distilled water and placed in orbital shaker for incubation at 125 rpm. The bacterial suspensions were diluted upto 10^-6^. Each dilution (100 mL) was spread onto petri plates containing Yeast Extract Mannitol (YEM) agar medium. Petri plates were incubated at 29°C for 96 hours and the colonies were counted on colony counter and expressed in colony forming unit (CFU) (Bhuyan et al., 2023).

### 2.3 Molecular characterization of drought tolerant rhizobacteria

Out of 12 isolates, the two most potent bacterial isolates were selected to perform 16S RNA gene sequencing. Raw DNA of the isolates was extracted (Chèneby et al., 2004) and subjected to amplification. For polymerase chain reaction, PCR system was programed as primarily denaturation at 94°C for 4 minute and 30 sec followed by 30 rounds. Each round with denaturation at 94°C for 0.5 min, annealing at 57°C for 0.5 min and extension at 68°C for 1.5 min. Amplified PCR products (with no fluorescent primers) were purified using QIA quick PCR purification kit (QIAGEN) following to the standard protocol recommended by the manufacturer and sent for sequencing. Gene sequences of bacterial isolates MJ1 and SRJ4 for 16S RNA gene were compared with the common sequences of the Gene Bank databases using Blast Tool. The sequences were deposited in the GenBank with accession number MT367716 (*Bacillus subtilis*), MT367717 (*Curtobacterium citreum*) for SRJ4 and MJ1, respectively.

### 2.4 Experimental design and growth condition

#### 2.4.1 Inoculum preparation

Out of twelve PGPR isolates, two most efficient PGP bacterial isolates with superior root colonization and viable cell count were selected for pot experiment. One of the bacteria (SRJ4) has good biofilm production potential while the other one (MJ1) was relatively weaker biofilm producer. Bacterial inoculum of selected bacterial isolates was prepared in 250 mL broth and incubated (28 ± 2°C; 48 hr). Incubated cultures were centrifuged (6000 rpm; 5 min) and pellets of bacterial cells were collected. These pellets were re-suspended in autoclaved distilled water. Cell density of the bacterium was maintained i.e., 10^8^–10^9^ CFU mL^-1^ by adjusting optical density at OD_600_ = 0.5. The inoculum of biofilm producing PGPR isolates was inoculated to sterilized maize seed.

#### 2.4.2 Experimental layout

Seeds of hybrid maize variety named Pioneer 30T60 were surface sterilized by dipping in 5% NaClO solution for 5 min and washed with 95% ethanol followed by four washings with autoclaved distilled water. The required volume of inoculum suspension was mixed with sterile peat (seed: peat; 4:1). Inoculated peat was mixed with sugar solution (10%) for seed dressing. To prepare uninoculated control treatment, sterilized peat mixed with sterilized broth and sugar solution (10%) was utilized for seed coating. Inoculated and uninoculated seeds were placed for drying in ambient laboratory conditions for 12 hr. Physico-chemical properties of soil used in this experiment were analyzed (**Table 1**). The soil used in this experiment was sterilized and the recommended level of N & P chemical fertilizers was applied as urea & diammonium phosphate (DAP), respectively. The soil field capacity was kept at water potential of -10 to -33 kPa. All the treatments were applied in triplicates. The pots were irrigated, and the 1^st^ irrigation was done after 7 days of seed germination. In the first set of maize plants, growth was maintained for 45 days and then irrigation was skipped, and plant samples were collected at the 1^st^, 3^rd^ and 6^th^ day of drought interval for evaluation of plant physiological and biochemical traits.

**Table 1:** Physicochemical properties of soil used in the study.

Parameter	Value
Texture of soil	Sandy clay loam
Bulk density (g cm^-3^)	1.40 ± 0.05
pH	7.50 ± 0.45
Electrical conductivity (dS m^-1^)	1.60 ± 0.05
N-contents (mg kg^-1^)	6.43 ± 0.35
Available P-contents (mg kg^-1^)	8.19 ± 0.25
Extractable K-contents (mg kg^-1^)	13.20 ± 0.26
WEOC (mg kg^-1^)	0.69 ± 0.02

In the second pot experiment, all the other arrangements were kept the same except for variation in moisture level. By determining soil water content (oven dry method), respective soil moisture level was calculated for 75% and 50% of the field capacity (FC). The levels of varying moisture involving 100%, 75% and 50% of FC in each treatment were maintained throughout pot trial, by adding water daily on weight loss basis relative to initially determined soil water content and soil water holding capacity. The soil moisture status of pots was checked thrice daily. The increase in plant weight was neglected while maintaining the required moisture level in pot experiment. Two levels of glycine betaine (i.e., 0 mM, 50 mM, respectively) were applied at 25, 50 days after sowing as spray. Growth parameters were determined after one week of second spray.

#### 2.4.3 Plant growth and physiological responses

Fresh and dry root biomass of maize plants was measured at all three drought intervals. Total P contents of ground plant samples (0.5 g) were analyzed after digesting plant samples with 5 mL of conc. H_2_SO_4_ and H_2_O_2_ (Wolf, 1982). The relative water content of leaf was determined following weatherley (1950) method. The water potential (-Mpa) of leaf was attained using Scholander pressure chamber (Scholander et al., 1965) and osmatic potential was measured by Capell and Doerffling (1993) method.

Photosynthetic pigments (chlorophyll a, chlorophyll b) were determined by following Arnon (1949) method. In briefs, 0.1 g of fresh leave was minced in 10 mL of 95.5% acetone solution and absolute ethyl alcohol with 1:1 ratio and kept in dark then concentration of all pigments was measured on UV- visible spectrophotometer under absorbance at 645 nm and 663 nm.

For determination of glutathione peroxidase (GPX), catalases (CAT) and ascorbate peroxidase (APX) activities, enzyme extract was obtained. Fresh samples of shoots (0.5 g) were minced in buffer (5 mL) and centrifuged to get supernatant as enzyme extract (Zhang and Kirkham, 1996; Bian and Jiang, 2009). To determine APX activity, 2 mM ascorbate was mixed in the enzyme extraction buffer. While the reaction-mixture for GPX was prepared having total volume of 3 mL by mixing phosphate buffer (50 mM; pH-7), 1% guaiacol (1 mL) and enzyme extracts (0.3 mL). The reaction began after the addition of 1% H_2_O_2_ (1 mL). Decrease in absorbance was noted at 420 nm (Upadhyaya et al., 1985). To measure APX activity, enzyme extracts (0.1 mL) were added to the reaction mixture having phosphate buffer (50 mM; pH-7), 1% H_2_O_2_ (0.2 mL), ascorbate (0.5 mM) and EDTA (0.1 mM) and reduction in absorbance was read at 240 nm (Chen and Asada, 1989). The CAT activity was determined by mixing enzyme extract and the reaction mixture prepared in phosphate buffer (50 mM; pH-7) and H2O2. Decrease in absorbance of H_2_O_2_ within 1 min was recorded at 240 nm (Maehlay and Chance, 1954).

#### 2.4.4 Real-time qPCR

To extract ribonucleic acid (RNA), Leaf samples in triplicate from each day and treatment, were ground in liquid nitrogen. Optimal RNA extract was obtained using extraction reagent (TRIzol, Invitrogen, USA). The concentration and purity of the extracted RNA was assessed by quantification using a Nanodrop spectrophotometer (Thermo Fisher Scientific, San Jose, USA). Genomic DNA contamination in RNA extracts was mitigated by DNase treatment. The cDNA synthesis was achieved using 1 µg of total RNA as a template, employing the high-capacity cDNA reverse transcription kit (Applied Biosystems, USA), followed by real-time PCR using SYBR Green Master Mix (Roche Applied Science, USA) and a real-time PCR system (Applied Biosystems). The PCR profile was set as, 50°C for 2 min and 95°C for 10 min; 95°C for 35 s and 60°C for 1 min repeated in 35 cycles and final extension at 72°C for 10 min. Sequences of the primers used in this study are listed in **Supplementary Table 2**. Actin gene was used as a reference for normalization, and transcript abundance was calculated and expressed as 2^(-ΔΔCT), facilitating the analysis of fold changes in transcription between inoculated and control plants across different harvest times and treatments (Livak and Schmittgen, 2001).

### 2.5 Data analysis

All statistical analyses were performed using SPSS software (Version 21). The effects of bacterial inoculations across induced moisture period for biochemical, molecular and physiological attributes were assessed using two-way ANOVA. The effect of moisture regimes in the presence and absence of glycine betaine on root and shoot growth and chlorophyll contents of inoculated and inoculated plants was also assessed by multiple ANOVA at 5% probability level. For comparison of triplicates, Tukey’s HSD *post hoc* test was employed.

## 3 Results

Qualitative analysis of different bacterial isolates showed varying potential for plant growth promotion. Characteristics of selected bacterial isolates under drought stress (-0.30 Mpa) are presented in **Supplementary Tables 1**, **2**. Pigment development in Indole acetic acid (IAA) production assay, halo zone formation (on tri-calcium phosphate (TCP) and rock phosphate amended media), and ACC deaminase activity were found positive for both bacterial isolates (SRJ4 and MJ1). However, bacterial isolates SRJ4 was good in exopolysaccharide secretion while MJ1 was found incapable of exopolysaccharide secretion. Similarly, biofilm formation showed higher values in SRJ4 whereas MJ1 presented weak expression of biofilm formation (**Table 2**).

**Table 2:** Characteristics of bacterial isolates under drought stress (-0.30 Mpa).

Bacterial Isolates	IAA Pigment development L-TRP (+)	P solubilization Halo zone on TCP	P solubilization Halo zone on Rock Phosphate	ACC deaminase activity	EPS secretion	Biofilm formation Absorbance at λ_600_
**SRJ4**	+++	+	+	+	+	3.392
**MJ1**	+++	+	+	+	–	0.235
**Control**	–	–	–	–	–	–

### 3.1 Auxin production assay

Data regarding production of Indole acetic acid (IAA) in the absence and presence of L- tryptophan precursor at varying levels of moisture contents (-0.05 to -1.61 Mpa) is presented in **Table 3**. At 0% PEG (-0.05 Mpa) having no L- tryptophan, 5.67 and 6.12 mg L^-1^ IAA was found in SRJ4 and MJ1, respectively. Similarly, 9.89 and 11.68 mg L^-1^ IAA was estimated in SRJ4 and MJ1, respectively with L- tryptophan in the reaction mixture. Maximum values of IAA production were observed at 4.16% PEG (-0.30 Mpa) in the absence/presence of L- tryptophan for both the bacterial isolates while minimum values were recorded at 20% PEG (-1.61 Mpa). The IAA production of SRJ4 ranges from 2.76 – 7.88 mg L^-1^ without L-tryptophan and 4.99 – 13.45 mg L^-1^ with L-tryptophan across all water stress levels whereas for MJ1, it ranged between 2.96 – 8.51 mg L^-1^ without L-tryptophan and 5.61 to 14.52 mg L^-1^ with L-tryptophan in reaction mixture. The IAA production even at 20% PEG level was better in MJ1 and SRJ4 isolates compared to other isolates therefore both the strains were selected for further evaluation.

**Table 3:** IAA production (mg L^-1^) by different isolates at varying levels of moisture stress (PEG levels) in the absence and presence of L- tryptophan precursor.

Bacterial Isolates	PEG Level=%; Water Potential= MPa													
	0.0% (-0.05MPa)		1.67% (-0.15MPa)		4.16% (-0.30MPa)		7.30% (-0.49MPa)		10.0% (-0.73 MPa)		15.0% (-1.14 MPa)		20.0% (-1.61MPa)	
	L-TRP (-)	L-TRP (+)	L-TRP (-)	L-TRP (+)	L-TRP (-)	L-TRP (+)	L-TRP (-)	L-TRP (+)	L-TRP (-)	L-TRP (+)	L-TRP (-)	L-TRP (+)	L-TRP (-)	L-TRP (+)
**SRJ4**	5.67 ± 0.09	9.89 ± 0.06	6.97 ± 0.12	11.91 ± 0.06	7.88 ± 0.05	13.45 ± 0.23	7.09 ± 0.08	11.84 ± 0.09	6.34 ± 0.10	10.59 ± 0.30	4.25 ± 0.18	7.16 ± 0.36	2.76 ± 0.05	4.99 ± 0.11
**MJ1**	6.12 ± 0.48	11.68 ± 0.32	7.53 ± 0.29	12.85 ± 0.33	8.51 ± 0.31	14.52 ± 0.28	7.66 ± 0.44	12.78 ± 0.79	6.80 ± 0.39	11.36 ± 0.52	4.56 ± 0.44	7.68 ± 0.63	2.96 ± 0.08	5.61 ± 0.21

### 3.2 Phosphate solubilizing assay and reduction in pH

**Figure 1:**
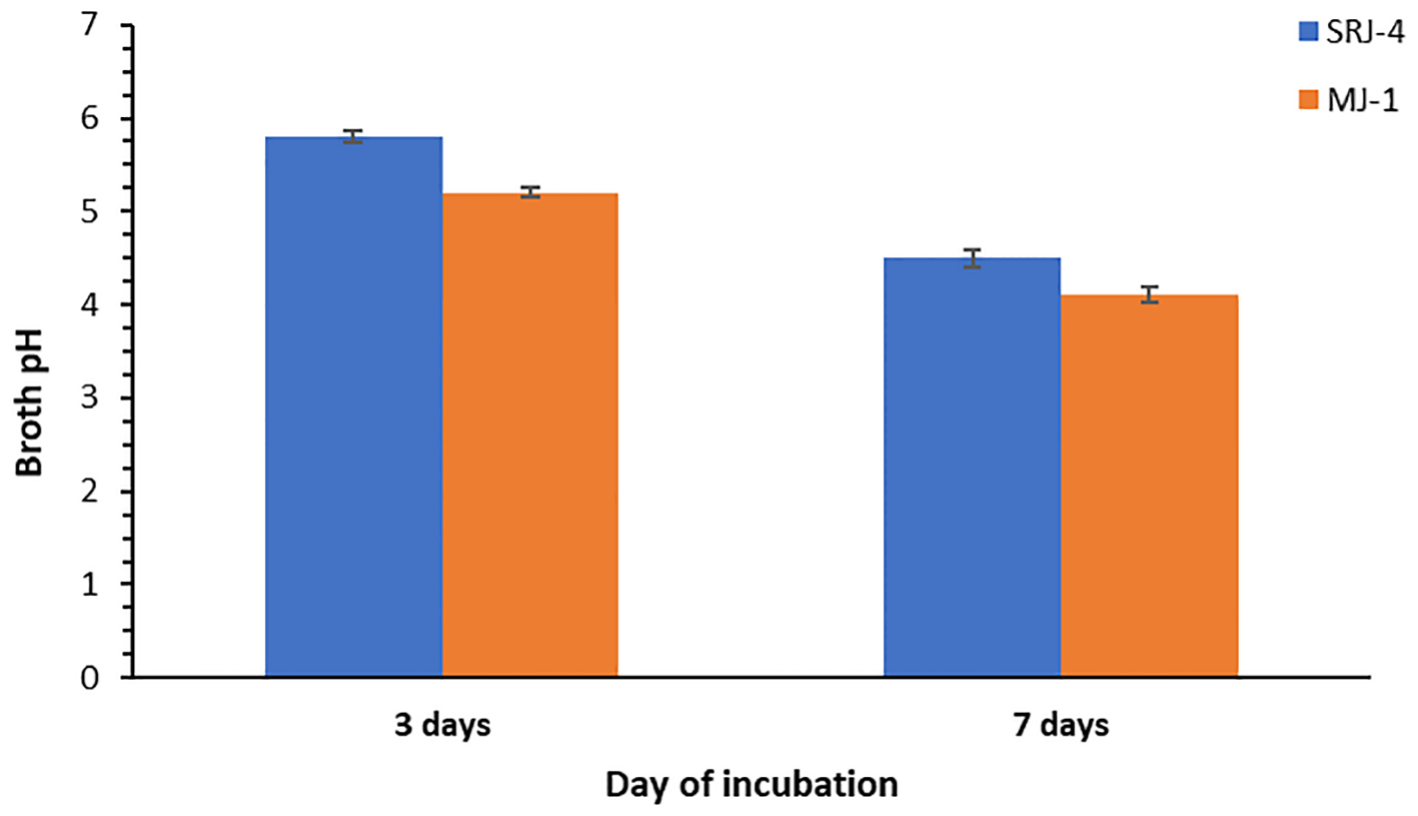
Reduction in pH of the growth medium (Pikovskaya's broth) after incubation of different drought tolerant isolates for 3 and 7 days at 28 °C.

Inoculation of bacterial isolates in Pikovskaya’s broth medium resulted in a decrease in pH showing the production of some organic acids by bacterial isolates. Observation taken after incubation of 3 days and 7 days, respectively is shown in **Figure 1**. Data regarding phosphate solubilization potential of different bacterial strains at varying levels of moisture stress is given in **Table 4**. Phosphate solubilizing for SRJ4 ranged between 576.34 to 295.96 mg L^-1^ while MJ1 presented a range of 640.34 to 345.72 mg L^-1^ across varying moisture stress level (-0.05 to -1.61 Mpa). Overall, both bacterial isolates showed gradual decrease in P solubilization activity with elevation of PEG concentration in broth culture. However, bacterial isolate MJ1 was characterized as a more efficient P solubilizer across all selected moisture stress levels (-0.05 to -1.61 Mpa) compared to SRJ4.

**Table 4:** Phosphate solubilization (mg L^-1^) of different isolates in broth solution with 0.5% rock phosphate at different PEG levels.

Bacterial Isolates	PEG Level=%; Water Potential= MPa						
	0.0% (-0.05MPa)	1.67% (-0.15MPa)	4.16% (-0.30MPa)	7.30% (-0.49MPa)	10.0% (-0.73 MPa)	15.0% (-1.14 MPa)	20.0% (-1.61MPa)
**SRJ4**	576.34 ± 16.89	553.29 ± 15.99	530.23 ± 13.83	461.07 ± 11.68	426.49 ± 12.79	386.15 ± 11.88	295.96 ± 10.94
**MJ1**	640.34 ± 19.20	614.73 ± 24.40	589.11 ± 17.56	512.27 ± 15.49	473.85 ± 18.16	429.03 ± 17.43	345.72 ± 13.37

### 3.3 Bacterial ACC-deaminase activity

The range of α-keto butyrate production for SRJ4 was found to be 45.8-332 µmol g^-1^ protein h^-1^ whereas, MJ1 showed ACC deaminase activity in the range of 39.8-280.1 µmol g^-1^ protein h^-1^ across varying selected levels of water potential (**Table 5**). ACC deaminase activity gradually increased with increasing water stress. Overall minimum values were recorded at 0% PEG (-0.05 Mpa) and highest values were recorded at 20% PEG (-1.61 Mpa) for both the isolates. Comparatively, SRJ4 was found imperious over MJ1 for ACC deaminase activity.

**Table 5:** ACC-deaminase activity (α-ketobutyrate µmol g^-1^ protein h^-1^) of different selected isolates at different PEG levels.

Bacterial Isolates	PEG Level=%; Water Potential= MPa						
	0.0% (-0.05 MPa)	1.67% (-0.15 MPa)	4.16% (-0.30 MPa)	7.30% (-0.49 MPa)	10.85% (-0.73 MPa)	15.0% (-1.14 MPa)	20.0% (-1.61 MPa)
**SRJ4**	45.8 ± 0.97	51.4 ± 1.07	123.6 ± 5.38	196.3 ± 7.52	267.9 ± 6.79	385.4 ± 12.67	332.8 ± 11.85
**MJ1**	39.8 ± 1.59	46.3 ± 1.79	134.4 ± 6.64	187.9 ± 6.93	279.3 ± 8.83	321.3 ± 9.49	280.1 ± 11.34

### 3.4 Root colonization ability under axenic conditions

Isolates SRJ4 and MJ1 showed maximum root colonization (CFU g^-1^ root) in well-watered conditions (0%PEG) and lowest values of root colonization were recorded at most water stressed plants (20% PEG). Bacterial isolate SRJ4 showed relatively improved root colonization across all water stress levels compared to MJ1across all the moisture stress levels as shown in **Table 6**.

**Table 6:** Root colonization (CFU g^-1^root) in maize plant by selected drought tolerant bacterial isolates at different PEG levels.

Bacterial Isolates	PEG Level=%; Water Potential= MPa						
	0.0% (-0.05MPa)	1.67% (-0.15MPa)	4.16% (-0.30MPa)	7.30% (-0.49MPa)	10.0% (-0.73 MPa)	15.0% (-1.14 MPa)	20.0% (-1.61 MPa)
**SRJ4**	6.14 x 10^6^	5.83 x 10^6^	5.65 x 10^6^	5.22 x 10^6^	4.6 x 10^6^	3.68 x 10^6^	2.15 x 10^5^
**MJ1**	5.68 x 10^6^	5.39 x 10^6^	5.22 x 10^6^	4.83 x 10^6^	4.26 x 10^6^	3.41 x 10^6^	1.99 x 10^5^

### 3.5 Physiological traits, genes expression and plant growth

**Figure 2:**
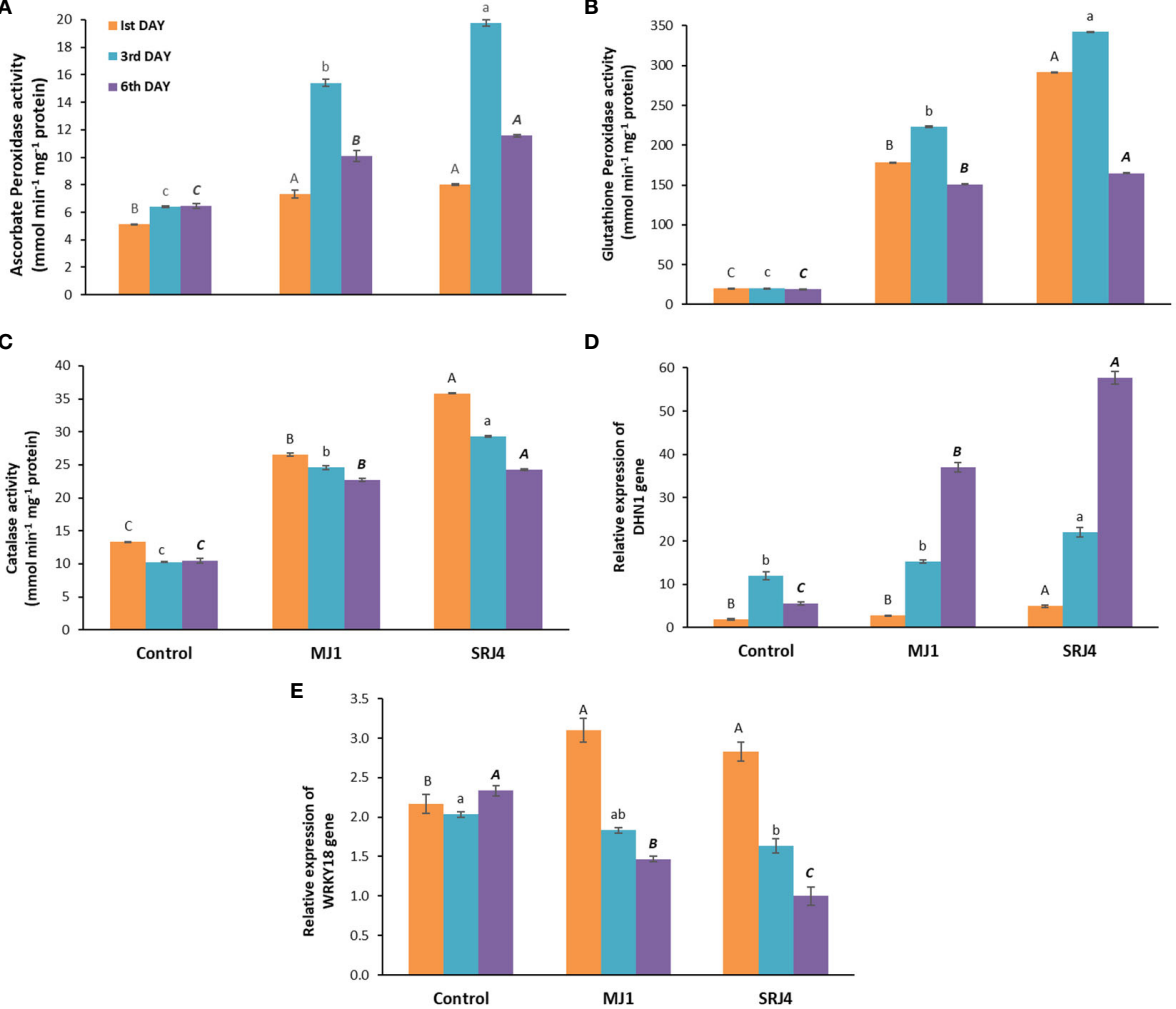
Effect of *Bacillus subtilis* (SRJ4) and *Curtobacterium citreum* (MJ1) at varying intervals of moisture stress on **(A)** ascorbate peroxidase activity, **(B)** glutathion peroxidase activity, **(C)** catalase activity **(D)** relative expression of DHN1 gene, **(E)** relative expression of WRKY18 gene of maize plants. Error bars are ± standard error of means (n = 3). Bars with different letters at each interval differ significantly from each other.

Contrasting activities of antioxidant enzymes were recorded on different days of moisture stress after skipping irrigation (1^st^, 3^rd^ and 6^th^ day) as presented in **Figures 2A-C**. Inoculation, period of moisture stress as well as their interaction proved to have significant effects on antioxidant enzymes activities as presented in **Supplementary Table 3**. Overall, the lowest ascorbate peroxidase activity was found in uninoculated control plants while plants treated with bacterial strain MJ1 and SRJ4 showed significantly higher ascorbate peroxidase activity. Bacterial isolate SRJ4 showed relatively enhanced activity at all three harvest times compared to that of MJ1. Moreover, maximum activity was recorded at 3^rd^ day of moisture stress among inoculated and uninoculated plants.

Significantly low activity of glutathione peroxidase (GPX) was observed in uninoculated control plants at all three harvests compared to respective inoculated plants. Bacterial isolate SRJ4 showed significantly enhanced activity compared to that of MJ1 and uninoculated control plants. Among all the three harvests, relatively more extent of enzyme activity was expressed by the plants at Ist and 3^rd^ days of moisture stress. Bacterial isolate MJ1 showed higher GPX activities at 1^st^, 3^rd^ and 6^th^ days of moisture stress (8, 10 and 7.9 folds) than control plants. Similarly, SRJ4 showed 14, 16, and 8 folds higher GPX activity at 1^st^, 3^rd^ and 6^th^ days of moisture stress, respectively, compared to that of control plants. Highly stimulated catalase activity was determined SRJ4 and MJ1 inoculated maize plants compared to uninoculated plants. Plants inoculated with strain MJ1 showed 2, 2.4 and 2.2 folds more CAT activity at 1^st^, 3^rd^ and 6^th^ day of drought stress, respectively compared to uninoculated control plants. While SRJ4 proved to be superior for CAT activity with 2.6, 2.8 and 2.3 folds higher activity at 1^st^, 3^rd^ and 6^th^ day of drought stress, respectively over uninoculated control plants. Among all the harvest times, maximum values of CAT activity were found at 1^st^ day of drought stress which gradually decreased with time.

The investigation into the relative transcript abundance of drought-responsive genes in maize plants revealed a gradual and substantial up-regulation of the DHN1 gene under increasing drought stress intervals (3-6 days) in both inoculated and un-inoculated plants. Notably, SRJ4 inoculation demonstrated a higher expression level compared to MJ1, indicating a differential response to the inoculation treatments. In contrast, un-inoculated control plants did not exhibit a clear trend in DHN1 gene expression under varying stress conditions. In control plants, DHN1 gene expression showed an initial increase at the 3^rd^ day of moisture stress, followed by a decline at the 6^th^ day, highlighting the dynamic nature of gene regulation in response to moisture stress (**Figure 2D**).

The WRKY18 gene in un-inoculated control plants displayed differential expression across all stress intervals, with downregulation at the 3^rd^ day and up-regulation at the 6^th^ day compared to the 1^st^ day of moisture stress. Inoculated plants, however, exhibited downregulation of WRKY18 gene expression at both the 3^rd^ and 6^th^ day compared to the 1^st^ day of drought stress, with MJ1-inoculated plants showing relatively higher expression than SRJ4. Moreover, a reduced basal level of WRKY18 transcription was observed in inoculated plants compared to uninoculated control plants under drought stress, suggesting a potential impact of inoculation on gene regulation in response to drought (**Figure 2E**).

**Figure 3:**
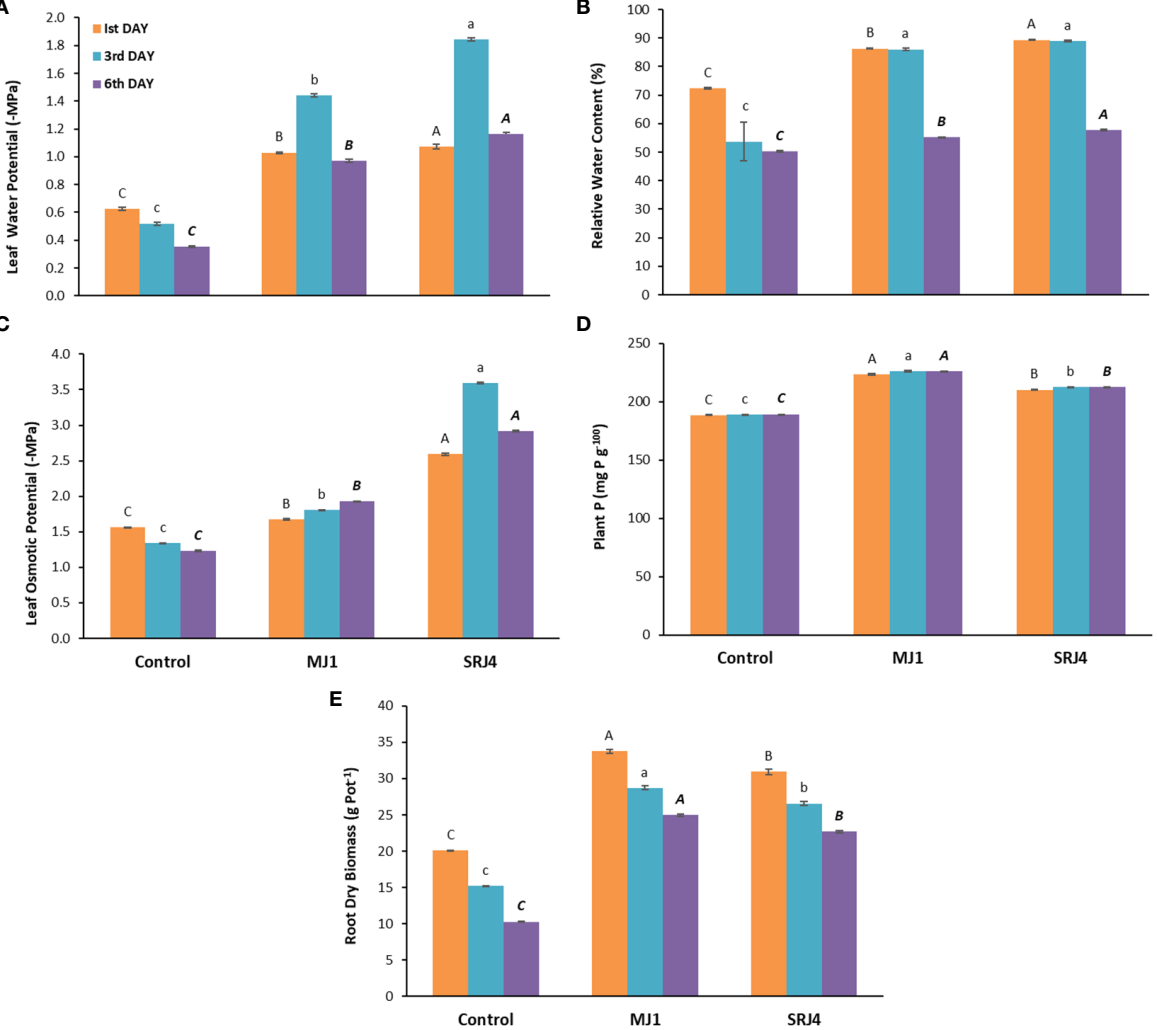
Effect of *Bacillus subtilis* (SRJ4) and *Curtobacterium citreum* (MJ1) at varying intervals of moisture stress on **(A)** Leaf water potential, **(B)** relative water content, **(C)** leaf osmotic potential, **(D)** plant P content and **(E)** root dry biomass of the maize plants. Error bars are ± standard error of means (n = 3). Bars with different letters at each interval differ significantly from each other.

Leaf water potential of inoculated plants was found higher compared to that of uninoculated plants at all three harvests (**Figure 3A**). Relatively higher water potential was determined at the 3^rd^ day of drought stress. The result regarding relative water content at 1^st^ day of drought stress showed 19 and 23% increase in MJ1 and SRJ4 inoculated plants, respectively over that of uninoculated control. At 3^rd^ and 6^th^ day of drought stress, 1.6 and 1.1 folds higher relative water contents were observed in MJ1 similarly, 1.7 and 1.1 folds higher relative water contents were recorded in SRJ4 inoculated plants at 3^rd^ and 6^th^ day of drought stress, respectively over uninoculated control (**Figure 3B**). It is noticeable that relative water content at 1^st^ and 3^rd^ day of drought stress were higher and comparable in inoculated plants compared to 6^th^ day of drought stress.

Data regarding osmotic potential showed a significant increase in osmotic potential of inoculated plants compared to uninoculated control plants (**Figure 3C**). Overall SRJ4 inoculated plants showed higher values especially at 3^rd^ day of drought stress compared to other inoculated and uninoculated plants. Noticeably, leaf osmotic pressure gradually decreased with time in uninoculated plants while it gradually increased in MJ1 inoculated plants with time while SRJ4 inoculated plants showed differential response with higher value at 3^rd^ day of drought stress.

Regarding total P contents, 18.57, 19.50 and 19.52% increase over uninoculated control plants was observed in MJ1 similarly, SRJ4 showed 11.5, 12.31 and 12.37% increase over uninoculated control at 1^st^, 3^rd^ and 6^th^ days of drought stress, respectively. MJ1 proved to be superior over SRJ4 for phosphorus uptake under drought stress conditions (**Figure 3D**). Dry root biomass data showed ascendancy of MJ1 over SRJ4 inoculation. Overall, inoculated plants produced more root dry biomass compared to uninoculated control which gradually decreased with time (**Figure 3E**).

Regarding findings of second pot experiment, multiple analysis of variance (MANOVA) denotes significantly different values of all the observed growth parameters at varying moisture levels (50%, 75% and 100% FC) and different inoculation treatments of bacterial isolates (**Supplementary Table 4**). Similarly, glycine betaine induced significant effects on observed growth parameters except root dry and fresh weight. Interaction of moisture levels and bacterial inoculation was effective for shoot fresh weight and dry weight, shoot length and root dry weight. Interaction of moisture level and glycine betaine worked efficiently on all observed growth parameters except root dry weight. Bacterial inoculation and glycine betaine did not produce any significant effect on any of the observed growth parameters. Interaction of glycine betaine, moisture level and bacterial inoculation was found to have positive influence on all observed plant growth parameters except root dry and fresh weight.

In depth statistical analysis of data regarding root length, root dry weight, shoot length and shoot fresh weight showed significant difference among bacterial treatments at 50% FC with no glycine betaine application. At 75% FC root fresh weight, root length, shoot fresh weight and shoot dry weight were found significantly different among bacterial treatments. At 100% FC, root fresh weight and shoot fresh weight was found significantly different by inoculating maize plants with different bacterial isolates (**Tables 7**, **8**).

**Table 7:** Effect of glycine betaine and bacterial inoculation on maize plant roots at varying moisture conditions.

Glycine Status	Moisture Levels	Bacterial Inoculation	Root length (cm)	Root fresh weight(g)	Root dry weight (g)
without Glycine betaine	**50% Field capacity**	**Control**	10.47 ± 22 a	1.43 ± 0.03 a	0.29 ± 0.01 b
		**SRJ4**	9.83 ± 0.58 a	1.43 ± 0.14 a	0.58 ± 0.08 a
		**MJ1**	14.13 ± 1.52 a	1.33 ± 0.13 a	0.41 ± 0.02 ab
		**SRJ4+MJ1**	11.70 ± 1.02 a	1.63 ± 0.14 a	0.36 ± 0.04 ab
	**75% Field capacity**	**Control**	13.63 ± 0.69 ab	1.20 ± 0.11 b	0.34 ± 0.02 a
		**SRJ4**	16.00 ± 1.76 a	1.84 ± 0.03 a	0.47 ± 0.06 a
		**MJ1**	15.73 ± 1.71 a	1.47 ± 0.09 ab	0.45 ± 0.06 a
		**SRJ4+MJ1**	9.13 ± 1.05 b	1.84 ± 0.08 a	0.41 ± 0.042 a
	**100% Field capacity**	**Control**	11.00 ± 0.45 a	2.37 ± 0.04 b	0.56 ± 0.06 a
		**SRJ4**	14.03 ± 0.92 a	2.70 ± 0.10 a	0.56 ± 0.06 a
		**MJ1**	13.63 ± 1.02 a	2.30 ± 0.05 b	0.64 ± 0.05 a
		**SRJ4+MJ1**	13.00 ± 0.75 a	2.43 ± 0.03 ab	0.49 ± 0.01 a
With Glycine betaine	**50% Field capacity**	**Control**	10.97 ± 0.96 a	1.33 ± 0.15 a	0.34 ± 0.03 b
		**SRJ4**	15.23 ± 1.92 a	2.05 ± 0.31 a	0.60 ± 0.08 a
		**MJ1**	13.73 ± 1.63 a	1.77 ± 0.07 a	0.45 ± 0.02 ab
		**SRJ4+MJ1**	9.97 ± 0.73 a	1.53 ± 0.07 a	0.38 ± 0.02 b
	**75% Field capacity**	**Control**	10.23 ± 0.98 a	0.93 ± 0.18 b	0.41 ± 0.07 a
		**SRJ4**	11.00 ± 0.45 a	1.40 ± 0.21 ab	0.47 ± 0.05 a
		**MJ1**	10.23 ± 0.63 a	1.43 ± 0.09 ab	0.43 ± 0.04 a
		**SRJ4+MJ1**	10.13 ± 0.58 a	1.77 ± 0.07 a	0.41 ± 0.01 a
	**100% Field capacity**	**Control**	12.13 ± 0.96 a	1.87 ± 0.18 a	0.50 ± 0.06 a
		**SRJ4**	12.03 ± 1.03 a	2.03 ± 0.23 a	0.47 ± 0.06 a
		**MJ1**	11.40 ± 0.55 a	1.85 ± 0.27 a	0.38 ± 0.03 a
		**SRJ4+MJ1**	12.80 ± 0.89 a	2.40 ± 0.15 a	0.58 ± 0.05 a

Error bars are ± standard error of means (n = 3). Values with different letters among different inoculations at each water stress level differ significantly from each other.

**Table 8:** Effect of glycine betaine and bacterial inoculation on maize plant shoots at varying moisture conditions.

Glycine Status	Moisture Levels	Bacterial Inoculation	Shoot length(cm)	Shoot Fresh weight (g)	Shoot dry weight (g)
without Glycine betaine	**50% Field capacity**	**Control**	57.27 ± 1.15 b	9.57 ± 0.19 b	1.57 ± 0.06 a
		**SRJ4**	68.17 ± 1.17 a	14.13 ± 1.29 a	2.15 ± 0.30 a
		**MJ1**	70.40 ± 3.27 a	11.50 ± 0.81 ab	2.02 ± 0.16 a
		**SRJ4+MJ1**	73.00 ± 0.93 a	12.77 ± 0.67 ab	2.24 ± 0.14 a
	**75% Field capacity**	**Control**	54.87 ± 2.07 a	9.67 ± 0.54 a	1.58 ± 0.05 b
		**SRJ4**	65.70 ± 2.60 a	9.07 ± 0.42 a	1.83 ± 0.16 b
		**MJ1**	64.07 ± 3.34 a	11.9 ± 1.00 a	2.89 ± 0.31 a
		**SRJ4+MJ1**	61.17 ± 3.36 a	13.20 ± 1.56 a	2.33 ± 0.25 ab
	**100% Field capacity**	**Control**	55.27 ± 0.37 a	11.93 ± 1.55 ab	1.93 ± 0.09 a
		**SRJ4**	62.63 ± 4.07 a	18.07 ± 0.73 a	2.13 ± 0.42 a
		**MJ1**	60.90 ± 3.65 a	11.10 ± 0.66 b	1.95 ± 0.10 a
		**SRJ4+MJ1**	63.80 ± 2.05 a	16.50 ± 2.13 ab	2.55 ± 0.34 a
With Glycine betaine	**50% Field capacity**	**Control**	62.80 ± 3.90 ab	11.43 ± 1.28 a	1.75 ± 0.23 a
		**SRJ4**	73.06 ± 1.24 a	14.40 ± 0.92 a	2.26 ± 0.16 a
		**MJ1**	72.60 ± 2.67 a	14.63 ± 2.78 a	1.87 ± 0.37 a
		**SRJ4+MJ1**	54.66 ± 6.06 b	9.13 ± 0.52 a	1.75 ± 0.34 a
	**75% Field capacity**	**Control**	65.00 ± 1.36 b	8.87 ± 0.34 a	2.16 ± 0.13 b
		**SRJ4**	80.13 ± 4.31 a	14.27 ± 2.03 a	2.94 ± 0.06 a
		**MJ1**	82.83 ± 3.78 a	10.13 ± 5.37 a	3.16 ± 0.18 a
		**SRJ4+MJ1**	82.77 ± 1.66 a	17.33 ± 0.84 a	2.77 ± 0.15 ab
	**100% Field capacity**	**Control**	67.47 ± 0.47 a	18.73 ± 1.09 b	2.97 ± 0.40 a
		**SRJ4**	74.13 ± 0.75 a	24.20 ± 0.75 ab	3.30 ± 0.14 a
		**MJ1**	69.07 ± 2.83 a	22.13 ± 1.01 bc	3.26 ± 0.11 a
		**SRJ4+MJ1**	75.50 ± 2.57 a	26.83 ± 2.18 a	3.95 ± 0.27 a

Error bars are ± standard error of means (n = 3). Values with different letters among different inoculations at each water stress level differ significantly from each other.

Glycine betaine application minimized the difference among all the observed growth parameters except shoot length and root dry weight under varying applications of bacterial isolates at 50% FC. At 75% FC, root fresh weight shoot length and shoot dry weight produced significant differences among bacterial inoculated treatments. At 100% FC, only shoot fresh weight and shoot length were significantly different from each other’s (**Tables 7**, **8**). Overall, SRJ4 proved better compared to MJ1 for growth attributes under drought stress. Surprisingly, consortia of SRJ4 and MJ1 showed a slight decrease in growth output compared to sole application of bacterial inoculants at water stress condition. Moreover, growth response in consortia inoculated plants was comparatively higher than uninoculated control plants. More obvious differences among consortia and sole application of bacteria inoculants were observed for growth attributes especially at 50% FC compared to 75 and 100% FC.

**Figure 4:**
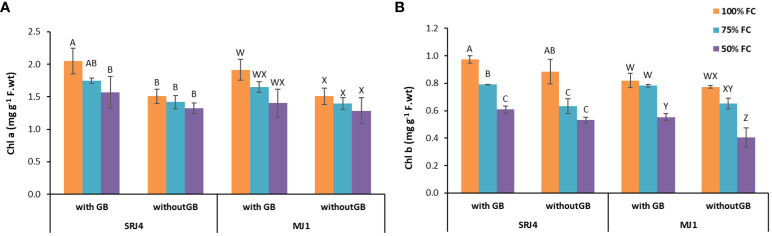
Effect of *Bacillus subtilis* (SRJ4) and *Curtobacterium citreum* (MJ1) and glycine betaine at varying levels of moisture stress on **(A)** chlorophyll a and **(B)** chlorophyll b content of the maize plants. Error bars are ± standard error of means (n = 3). Bars with different letters differ significantly from each other within each inoculation.

Statistical analysis of chlorophyll contents revealed significant influence of moisture stress on chlorophyll a chlorophyll b (**Figures 4A, B**), total chlorophyll (a+b) and chlorophyll a/b ratio (**Supplementary Figure 1**). Bacterial inoculation, however, significantly changed only chlorophyll b content. Application of glycine betaine also played a substantial role in chlorophyll a, b and total chlorophyll production. Interactions of drought stress x bacterial inoculation x glycine betaine application did not produce significant change in the observed parameters (**Supplementary Table 4**).

For chlorophyll a, bacterial inoculant SRJ4 showed significant difference among all three moisture stress levels of glycine betaine sprayed and non-sprayed plants. Similarly, MJ1 inoculated plants showed considerable difference among moisture stress levels and glycine betaine applications (**Figure 3**). Chlorophyll b and total chlorophyll and chlorophyll a/b also showed similar differences among different applications of moisture stress and glycine betaine. It is notable that chlorophyll contents were higher in glycine betaine sprayed plants compared to non-sprayed plants in both SRJ4 and MJ1 inoculated plants (**Table 5**).

## 4 Discussion

It is well known fact that plants are constantly subjected to abiotic stresses including salinity, floods, temperature, drought, etc. preceding to loss or inadequate agricultural productivity (Umar et al., 2022). Drought may trigger substantial productivity decline in semi-arid and arid zones where food production is entirely dependent on precipitation (Seleiman et al., 2021). The involvement of rhizobacteria in nutrient regulation and disease management is well recognized, but their function of managing abiotic stress is now acquiring attention worldwide (Yang et al., 2009). A rhizobacteria exhibiting plant growth promotion properties under stress conditions may prove to be efficient inoculants for stressed crop production systems. The drought tolerant phosphate solubilizing rhizobacteria can serve as proficient tool to improve crop yield in water and nutrient deficient lands and may consequently, expand agricultural system to ensure food security. The current study investigated the potential of two bacterial strains; *Bacillus subtilis* SRJ4 (Accession # MT367716) and *Curtobacterium citreum* MJ1 (Accession # MT367717) to alleviate the drought related stress of different levels on maize plants. The PGP bacteria with additional characteristic of stress tolerance not only mitigated the negative impacts of changing climate but also developed a proficient plant microbe interaction to improve plant growth.

In the current investigation, a total of 12 out of 40 purified isolates were characterized, those were isolated from maize rhizosphere of various native drylands (data not presented). All the isolates were evaluated for their drought tolerance ability for varying moisture stress levels using PEG6000 (-0.05 Mpa to -1.16 Mpa) and screened for their PGP traits under drought stress. In our study, two most efficient strains (MJ1 and SRJ4) those were able to grow at maximum negative water potentials of -1.16 Mpa having remarkably higher phosphate solubilization, IAA and ACC deaminase production under drought stress, were pursued. Many scientific groups have already reported better root growth, improved water and nutrient uptake by inoculating IAA producing bacteria under drought stress (Dimkpa et al., 2009; Vurukonda et al., 2016). Likewise, phosphate solubilizing rhizobacteria were also reported helpful in drought stress alleviation in plants (Shintu and Jayaram, 2015). Further, PGP rhizobacteria that have ACC-deaminase activity can assist plants to endure both biotic and abiotic stress (Ali et al., 2014). All these characteristics including biofilm formation, IAA production, ACC deaminase activity, P solubilization potential, and root colonization ability in both selected isolates of the current study under drought stress and control conditions were exploited by setting pot experiments.

Indole acetic acid (IAA) producing bacteria may exert positive effect on plant growth when inoculated or grow in the vicinity of rhizosphere. IAA is physiologically the most active member of auxin hormone. The rhizospheric bacteria usually secrete IAA as secondary metabolites in the presence of surplus substrate exudates secreted by plant roots. Moreover, plant growth promotion depends upon the concentration of IAA. Low concentrations promote root growth while high IAA amounts inhibit plant development (Dilfuza, 2011). Many studies have been carried out to evaluate the IAA secretion potential of drought resistant bacteria (Kaur et al., 2015; Yasmin et al., 2017). Bacterial isolates screened in the current study showed differential potential to form IAA from tryptophan precursor. Higher levels of IAA production at water potential -1.16Mpa (20%PEG) were detected in bacterial isolates MJ1, SRJ4. Moreover, the bacterial ability to increase the IAA production with increasing levels of osmotic stress with the addition of PEG in the growth medium is counted as osmotic tolerance (Marulanda et al., 2009).

Drought can minimize plant growth and development by limiting soil ‘P’ uptake (Cramer et al., 2009). The phosphate solubilizing bacteria solubilize ‘P’ by the secretion of organic acid that act as chelating agent. Organic acids reduce the pH of solution by providing the source of protons (Marhual et al., 2011). It is observed that release of organic acid leads to lowering of pH which is main cause of solubilization of inorganic phosphate (Marra et al., 2015). It is also reported that the effective solubilization of phosphate in acidic soil is because of the production of lactic acid, oxalic acid and citric acids (low molecular weight) that are involved in the solubilization of phosphate. Many bacterial species including *Curtobacterium* and *Bacillus* sp. are known for their potential to secrete organic acids and plant growth promotion under drought stress and metal toxicity. We got similar findings during the present investigation as reported by Zhang et al. (2021).

Explicit rise in ACC concentrations and subsequently, ethylene production under drought stress have frequently been stated in plants since long time. The inhibitory effects of ethylene induced by drought stress might have been eliminated through ACC-deaminase activity of the PGPR. It was also noted that ACC-deaminase producing PGPR can improve water use efficiency in inoculated peas under moisture stress (Mayak et al., 2004). As inoculation with PGPR having ability to produce ACC deaminase deliberates endurance against drought stress in different plants, so the bacterial isolates with good ACC deaminase activity obtained in this study could be inoculated to drought stressed plants to cope up the moisture deficient conditions. Bacterial isolates assayed in the current study have prime ability to reduce endogenous levels of ethylene by hydrolyzing ACC-deaminase into α-ketobutyrate. Selected isolates showed gradual decrease in ACC deaminase activity in lab conditions and reduced stress on plants under drought condition. In the current investigation both the inoculants have good ACC deaminase activity resulting increase in root elongation and biomass production and these findings are in agreement with that of Magnucka and Pietr (2015) and Chandra et al. (2018) showing improvement in root and shoot growth of wheat and finger, respectively.

Development of biofilm by certain PGPRs has been recognized as an important character for effective survival mechanism in rhizosphere under stressed conditions (Ansari and Ahmad, 2019). In our study, various bacterial isolates exhibited differential potential to produce biofilm and exopolysaccharide secretion which was exploited to assess their potential contribution to help plants under water stressed conditions. It was obvious from the results that high biofilm producing SRJ4 bacteria induced a strongly positive influence on maize plant growth compared to relatively low biofilm producing MJ1 bacteria. It is also notable that when both bacteria were inoculated in combination, the results slightly reduced as compared to inoculation of sole SRJ4 bacteria. That might be due to the diverse nature of bacterial growth requirements under certain environmental conditions. In co-inoculation of different bacterial strains there must be synergistic relationship between the co-inoculated bacteria to obtain increase in plant biomass compared to uninoculated control plants. Bacterial inoculant in the current investigation showed significantly varying response in the absence of glycine betaine. However, in the presence of glycine betaine all the bacterial inoculations produced non significantly different response that was higher than uninoculated control.

In the current study, bacterial inoculation confirmed notably excessive relative water content (RWC) than uninoculated control. Abscisic acid accumulation is one of the most obvious rejoinders of plants to drought stress. Abscisic acid plays a vital function in plant moisture regulation under moisture deficit environment by stimulating signaling cascade of stomatal closure to minimize water loss (Munemasa et al., 2015). The acquired results of current study are in line with the findings of other experiments which indicate that the PGPR inoculation helps in maintenance of RWC and transpiration that finally induce drought tolerance (Sandhya et al., 2009; Sandhya et al., 2010; Ahmad et al., 2022). Glycine betaine also induces positive impact on plant growth and water retention under water stress conditions as observed by Ashraf and Follad (2007). Drought restricts plant growth by decreasing cell division and cell enlargement, consequently reduction in root and shoot growth occurs (Anjum et al., 2011a; Claeys and Inzé, 2013). We observed the same trend of root and shoot growth reduction under drought stress in our current investigation. However, like all other parameters, a significant increase in osmotic potential, water potential and root dry biomass was observed in glycine betaine sprayed inoculated plants compared to uninoculated control plants.

Previous studies have also revealed that balance between antioxidant enzymatic activities and distribution of reactive oxygen species (ROS) in tissues is very important. Drought and oxidative stress are complex and interconnected phenomena showing analogous cellular responses including enzymatic and non-enzymatic antioxidants activities and accumulation of compatible solutes (Cia et al., 2012). During moisture deficient conditions, increased level of ROS intensifies oxidative stress due to the accumulation of O_2_^–^ and H_2_O_2_ in chloroplasts, mitochondria, and peroxisomes. The ability of plants to overcome the effect of oxidative stress depends partially on the stimulation of antioxidant enzymes like catalases (CAT) and different species of peroxidases (Alscher et al., 2002). Though, plant cells have different antioxidant enzymes having ability to eradicate the reactive free radicals or suppress their formation (Simova-Stoilova et al., 2008) yet, inoculation of drought tolerant PGP bacteria may up-regulate this ability in the inoculated plants. This may be due to the less accumulation of H_2_O_2_ and O_2_^-^ in plant mitochondria and chloroplasts. In the present study, augmented CAT activity in bacterial inoculated plants under drought stress suggests that these bacteria can be used to lessen the oxidative damage, provoked by moisture deficit. Similar findings were reported by Sarker and Oba (2018) and Kohler et al. (2008) during their investigation on water stressed plants. In the current investigation we also observed upregulated expression of DHN1 (gene respond to water stress) while down regulated expression of WRKY18 gene (transcription factor gene of detoxification) in inoculated maize plants with progressive increase in drought interval. Similar trend of drought stress responsive gene expression was reported by Ahmad et al. (2019) in drought stressed inoculated and uninoculated maize plant The characterization of regulatory genes in signaling pathways related to plant-rhizobacterial interactions is seen as a key step in developing targeted strategies to improve these interactions. This could have applications in agriculture, where optimizing the symbiotic relationship between plants and rhizobacteria can lead to improved crop yield, resilience, and sustainability. Understanding the regulatory genes in signaling pathways allows researchers to develop strategies, possibly through genetic manipulation, breeding programs, or other interventions, to optimize plant-rhizobacterial interactions.

Regarding photosynthetic machinery, it is well known fact that chlorophyll contents reduce under the water stress conditions due to modulation in chlorophyll synthesis, stomatal closure and reduction in leaf conductance (Wang et al., 2010; Hasan et al., 2018). Production of ROS, lipid peroxidation and oxidative damage could also be some other potential reasons (Tátrai et al., 2016). Osmo protectants like glycine betaine help plants under stress conditions to withstand ROS induced enzyme damage, stabilize membrane integrity, and improve cell water retention in water stressed plants (Farooq et al., 2008). The reduced chlorophyll contents under water stress condition while improved chlorophyll contents using glycine betaine spray, are in line with the finding of Anjum et al. (2011b). Chlorophyll a content generally remains higher than chlorophyll b content in plants. Increase of chlorophyll contents in glycine betaine sprayed well-watered inoculated plants show good state of plant photosynthetic machinery as observed in the current investigation.

## 5 Conclusion

Present study establishes that the natural dryland soil provides habitat for various groups of potential drought tolerant PGPRs having differential potential of indole acetic acid production phosphate solubilization, ACC deaminase activity, exopolysaccharide secretion and biofilm formation. Our selected highly efficient bacterial isolates/inoculants having good PGPR characteristics and biofilm production potential increased root colonization of maize seedlings in a lab scale experiment. First pot experiment of the study illustrated stimulated antioxidants activity, water potential, relative water contents and osmotic potential in inoculated maize plants compared to uninoculated plants at all three drought stress intervals. Upregulation of drought stress responsive gene DHN1 and down regulation of WEKY18 gene was observed in inoculated drought stressed plants compared to uninoculated control plants. Plant P contents and dry biomass also indicated positive influence of bacterial inoculants under drought stress conditions compared to uninoculated control plants. Second pot experiment demonstrated improved plant growth and chlorophyll contents in inoculated maize plants in the presence of glycine betaine however, SRJ4 isolate proved superior over MJ1 both in the presence and absence of glycine betaine across all water stress levels.

## 6 Recommendations

Isolation of indigenous drought tolerant PGP bacterial isolates from stressed ecosystems could be used to develop bioformulations for the establishment of drought tolerance in stressed plants. Under field conditions, biosafety concerns can be addressed by using pigment-based fluorescent labeling of the introduced PGPR strains to validate their competitive ability against other soil microbes and subsequent root colonization potential, which are the most obvious features that address any biosafety concerns. The potential of drought tolerant microbial inoculants could be evaluated by integrated approach of applying these inoculants in combination with chemical or biochemical stress alleviators/osmoregulators.

## Data availability statement

The datasets presented in this study can be found in online repositories. The names of the repository/repositories and accession number(s) can be found below: Genbank accession number: MT367716 and MT367717.

## Author contributions

TY: Conceptualization, Funding acquisition, Methodology, Resources, Supervision, Writing – original draft, Writing – review & editing. MSA: Data curation, Formal analysis, Methodology, Software, Writing – review & editing. MT: Data curation, Formal Analysis, Investigation, Validation, Writing – original draft. SaA: Investigation, Methodology, Validation, Visualization, Writing – original draft. AS: Formal analysis, Investigation, Methodology, Validation, Writing – original draft. WH: Data curation, Formal analysis, Investigation, Methodology, Writing – original draft. MR: Investigation, Software, Visualization, Writing – review & editing. MH: Conceptualization, Formal Analysis, Software, Visualization, Writing – review & editing. AA: Conceptualization, Resources, Software, Writing – review & editing. ShA: Conceptualization, Resources, Software, Writing – review & editing.
