# Supplementary material for: Biofilm producing plant growth promoting bacteria in combination with glycine betaine uplift drought stress tolerance of maize plant
Source: Front Plant Sci. 2024 Feb 9;15:1327552. doi: 10.3389/fpls.2024.1327552 (PMC10884199; doi:10.3389/fpls.2024.1327552)
Supplement: Supplementary file 1 [file DataSheet_1.docx]

**S1 Characterization of bacterial strains for in vitro PGP traits**

| Characteristics | *Bacillus subtilis* (SRJ4) | *Curtobacterium citreum* (MJ1) |
| --- | --- | --- |
| Gram’s Test | + | + |
| Indole Acetic acid production | + | + |
| ACC deaminase | + | + |
| P solubilization | + | + |
| Biofilm formation | + | + |
| Catalase Test | + | + |
| Starch hydrolysis | + | + |
| Nitrate reductase test | + | - |
| Glycerol utilization | + | + |
| Sucrose utilization | + | + |
| Cellulase | + | + |
| Urease activity | + | - |
| Chitinase | + | + |
| Citrate utilization | + | - |
| Amylase | + | - |

**S2. List of maize genes with primer sequences used for real-time qPCR**

Gene name Forward (5’ - 3’) Reverse (5’ - 3’)

Actin TACCATGTTCCCTGGGATTG GTGGCGCAATCACTTTAACC

WRKY18 CGTGCCTACTGAAACATCGGA GTAAGCTCTAGGTGACGGGTTGTC

DHN1 GAAGGAGGAAGAAGGGAATCA GCTTCTCCTTGATCTTGTCCA

**S3. Two-way ANOVA for plant physiological and biochemical attributes at different moisture stress intervals and bacterial inoculations**

| **Source of Variance** | **Dependent Variable** | **Sum of Squares** | **df** | **Mean Square** | **F** |
| --- | --- | --- | --- | --- | --- |
| Drought stress interval | APX | 228.851 | 2 | 114.426 | 823.953*** |
|  | CAT | 168.745 | 2 | 84.373 | 646.021*** |
|  | GPX | 32090.36 | 2 | 16045.18 | 30602.19*** |
|  | RWC | 3959.075 | 2 | 1979.537 | 133.027*** |
|  | LWP | 0.982 | 2 | 0.491 | 1394.937*** |
|  | LOP | 0.435 | 2 | 0.218 | 839.157*** |
|  | RDB | 362.688 | 2 | 181.344 | 1257.812*** |
|  | Plant P | 20.652 | 2 | 10.326 | 22.477*** |
| Bacterial inoculation | APX | 239.533 | 2 | 119.766 | 862.41*** |
|  | CAT | 1631.609 | 2 | 815.805 | 6246.414*** |
|  | GPX | 283403.3 | 2 | 141701.6 | 270260.6*** |
|  | RWC | 2085.226 | 2 | 1042.613 | 70.064*** |
|  | LWP | 3.61 | 2 | 1.805 | 5129.811*** |
|  | LOP | 13.329 | 2 | 6.665 | 25706.33*** |
|  | RDB | 998.78 | 2 | 499.39 | 3463.8*** |
|  | Plant P | 6056.208 | 2 | 3028.104 | 6591.485*** |
| Drought stress interval x Bacterial inoculation | APX | 93.956 | 4 | 23.489 | 169.14*** |
|  | CAT | 70.83 | 4 | 17.707 | 135.581*** |
|  | GPX | 25993.03 | 4 | 6498.259 | 12393.81*** |
|  | RWC | 786.288 | 4 | 196.572 | 13.21*** |
|  | LWP | 0.588 | 4 | 0.147 | 417.963*** |
|  | LOP | 1.38 | 4 | 0.345 | 1330.429*** |
|  | RDB | 2.406 | 4 | 0.601 | 4.171* |
|  | Plant P | 4.212 | 4 | 1.053 | 2.292ns |

GPX = Glutathione peroxidase activity

CAT = Catalases activity

APX **=** Ascorbate peroxidase activity

RWC **=** Relative water content

LWP= Leaf water potential

LOP **=** Leaf osmatic potential

RDB **=** Root dry biomass

Plant P= Plant P content

**S4. Multivariate analysis of variance (MANOVA) for plant growth parameters at different moisture stress levels, bacterial inoculations and glycine betaine application**

| **Source of Variance** | **Dependent Variable** | **Sum of Squares** | **df** | **Mean Square** | **F** |
| --- | --- | --- | --- | --- | --- |
| Moisture Level | Root fresh weight (g) | 8.35 | 2 | 4.17 | 66.01** |
|  | Root length (cm) | 3.93 | 2 | 1.97 | 0.59ns |
|  | Shoot Fresh weight (g) | 717.40 | 2 | 358.70 | 43.11*** |
|  | Shoot length (cm) | 173.09 | 2 | 86.55 | 3.54* |
|  | Shoot dry weight (g) | 7.87 | 2 | 3.94 | 23.76*** |
|  | Root dry weight (g) | 0.15 | 2 | 0.08 | 9.72*** |
|  | Chlorophyll a | 0.74 | 2 | 0.37 | 5.524*** |
|  | Chlorophyll b | 0.69 | 2 | 0.34 | 61.129*** |
|  | Total chlorophyll (a+b) | 2.85 | 2 | 1.43 | 19.881*** |
|  | Chlorophyll a/b | 0.09 | 2 | 0.05 | 4.715** |
| Bacterial Inoculation | Root fresh weight (g) | 2.05 | 3 | 0.68 | 10.80*** |
|  | Root length (cm) | 60.44 | 3 | 20.15 | 6.01*** |
|  | Shoot Fresh weight (g) | 215.39 | 3 | 71.80 | 8.63*** |
|  | Shoot length (cm) | 1200.38 | 3 | 400.13 | 16.36*** |
|  | Shoot dry weight (g) | 4.04 | 3 | 1.35 | 8.13*** |
|  | Root dry weight (g) | 0.14 | 3 | 0.05 | 6.11*** |
|  | Chlorophyll a | 0.05 | 1 | 0.05 | 0.767ns |
|  | Chlorophyll b | 0.05 | 1 | 0.05 | 8.429** |
|  | Total chlorophyll (a+b) | 0.20 | 1 | 0.20 | 2.749ns |
|  | Chlorophyll a/b | 0.01 | 1 | 0.01 | 0.597ns |
| Glycine betaine Application | Root fresh weight(g) | 0.33 | 1 | 0.33 | 5.23* |
|  | Root length (cm) | 19.32 | 1 | 19.32 | 5.76* |
|  | Shoot Fresh weight (g) | 227.91 | 1 | 227.91 | 27.39*** |
|  | Shoot length (cm) | 1320.98 | 1 | 1320.98 | 54.02*** |
|  | Shoot dry weight (g) | 6.04 | 1 | 6.04 | 36.46*** |
|  | Root dry weight (g) | 0.00 | 1 | 0.00 | 0.22ns |
|  | Chlorophyll a | 0.90 | 1 | 0.90 | 13.36*** |
|  | Chlorophyll b | 0.10 | 1 | 0.10 | 18.534*** |
|  | Total chlorophyll (a+b) | 1.61 | 1 | 1.61 | 22.495*** |
|  | Chlorophyll a/b | 0.003 | 1 | 0.003 | 0.28ns |
| Moisture Level x  Bacterial Inoculation | Root fresh weight (g) | 0.66 | 6 | 0.11 | 1.73ns |
|  | Root length (cm) | 42.56 | 6 | 7.09 | 2.12ns |
|  | Shoot Fresh weight (g) | 138.24 | 6 | 23.04 | 2.77* |
|  | Shoot length (cm) | 342.86 | 6 | 57.14 | 2.34* |
|  | Shoot dry weight (g) | 3.20 | 6 | 0.53 | 3.22** |
|  | Root dry weight (g) | 0.15 | 6 | 0.02 | 3.13** |
|  | Chlorophyll a | 0.00 | 2.00 | 0.00 | 0.02ns |
|  | Chlorophyll b | 0.03 | 2.00 | 0.02 | 2.66ns |
|  | Total chlorophyll (a+b) | 0.04 | 2.00 | 0.02 | 0.29ns |
|  | Chlorophyll a/b | 0.01 | 2.00 | 0.01 | 0.56ns |
| Moisture Level x  Glycine betaine Application | Root fresh weight (g) | 1.22 | 2 | 0.61 | 9.61*** |
|  | Root length (cm) | 52.48 | 2 | 26.24 | 7.82*** |
|  | Shoot Fresh weight (g) | 231.44 | 2 | 115.72 | 13.91*** |
|  | Shoot length(cm) | 984.10 | 2 | 492.05 | 20.12*** |
|  | Shoot dry weight (g) | 5.16 | 2 | 2.58 | 15.58*** |
|  | Root dry weight (g) | 0.04 | 2 | 0.02 | 2.60ns |
|  | Chlorophyll a | 0.13 | 2.00 | 0.07 | 0.98ns |
|  | Chlorophyll b | 0.01 | 2.00 | 0.00 | 0.79ns |
|  | Total chlorophyll (a+b) | 0.09 | 2.00 | 0.05 | 0.65ns |
|  | Chlorophyll a/b | 0.04 | 2.00 | 0.02 | 1.99ns |
| Bacterial Inoculation x  Glycine betaine Application | Root fresh weight (g) | 0.19 | 3 | 0.07 | 1.02ns |
|  | Root length (cm) | 17.03 | 3 | 5.68 | 1.69ns |
|  | Shoot Fresh weight (g) | 5.87 | 3 | 1.96 | 0.24ns |
|  | Shoot length (cm) | 79.02 | 3 | 26.34 | 1.08ns |
|  | Shoot dry weight (g) | 0.34 | 3 | 0.11 | 0.68ns |
|  | Root dry weight (g) | 0.03 | 3 | 0.01 | 1.47ns |
|  | Chlorophyll a | 0.03 | 1.00 | 0.03 | 0.40ns |
|  | Chlorophyll b | 0.00 | 1.00 | 0.00 | 0.00ns |
|  | Total chlorophyll (a+b) | 0.03 | 1.00 | 0.03 | 0.36ns |
|  | Chlorophyll a/b | 0.00 | 1.00 | 0.00 | 0.46ns |
| Moisture Level x Bacterial Inoculation x  Glycine betaine Application | Root fresh weight (g) | 0.90 | 6 | 0.15 | 2.37* |
|  | Root length (cm) | 77.21 | 6 | 12.87 | 3.84** |
|  | Shoot Fresh weight (g) | 115.02 | 6 | 19.17 | 2.30ns |
|  | Shoot length(cm) | 630.91 | 6 | 105.15 | 4.30** |
|  | Shoot dry weight (g) | 0.80 | 6 | 0.13 | 0.80ns |
|  | Root dry weight (g) | 0.06 | 6 | 0.01 | 1.30ns |
|  | Chlorophyll a | 0.00 | 2.00 | 0.00 | 0.01ns |
|  | Chlorophyll b | 0.01 | 2.00 | 0.00 | 0.51ns |
|  | Total chlorophyll (a+b) | 0.01 | 2.00 | 0.00 | 0.04ns |
|  | Chlorophyll a/b | 0.00 | 2.00 | 0.00 | 0.19ns |


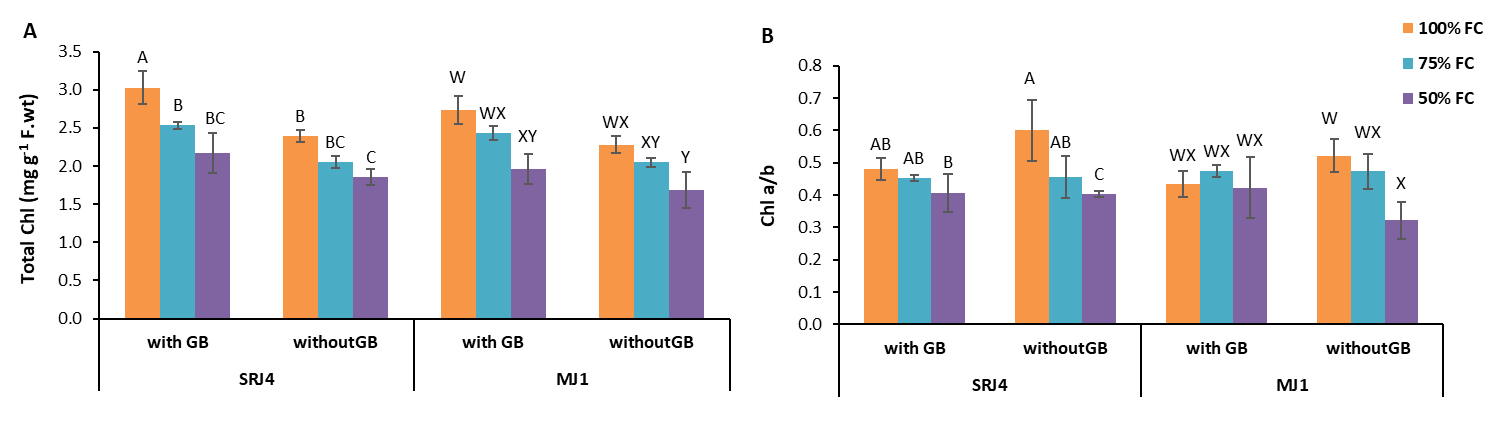


Figure S1

Effect of *Bacillus subtilis* (SRJ4) and *Curtobacterium citreum* (MJ1) and glycine betaine at varying levels of moisture stress on (A) total chlorophyll and (B) chlorophyll a/b ratio of the maize plants
